# Tartary Buckwheat Transcription Factor FtbZIP5, Regulated by FtSnRK2.6, Can Improve Salt/Drought Resistance in Transgenic *Arabidopsis*

**DOI:** 10.3390/ijms21031123

**Published:** 2020-02-07

**Authors:** Qi Li, Haixia Zhao, Xiaoli Wang, Jingyue Kang, Bingbing Lv, Qixin Dong, Chenglei Li, Hui Chen, Qi Wu

**Affiliations:** College of Life Science, Sichuan Agricultural University, No. 46, Xinkang Road, Ya’an 625014, China; lq760621985@gmail.com (Q.L.); zhaohaixia@sicau.edu.cn (H.Z.); wangxiaoli288@163.com (X.W.); kangjingyue2020@163.com (J.K.); lvbing1228@163.com (B.L.); dqxczwq00@163.com (Q.D.); lichenlei@sicau.edu.cn (C.L.); chenhui@sicau.edu.cn (H.C.)

**Keywords:** tartary buckwheat, AREB/ABF, salt and drought stress

## Abstract

bZIP transcription factors have been reported to be involved in many different biological processes in plants. The ABA (abscisic acid)-dependent AREB/ABF-SnRK2 pathway has been shown to play a key role in the response to osmotic stress in model plants. In this study, a novel bZIP gene, *FtbZIP5,* was isolated from tartary buckwheat, and its role in the response to drought and salt stress was characterized by transgenic *Arabidopsis*. We found that FtbZIP5 has transcriptional activation activity, which is located in the nucleus and specifically binds to ABRE elements. It can be induced by exposure to PEG6000, salt and ABA in tartary buckwheat. The ectopic expression of *FtbZIP5* reduced the sensitivity of transgenic plants to drought and high salt levels and reduced the oxidative damage in plants by regulating the antioxidant system at a physiological level. In addition, we found that, under drought and salt stress, the expression levels of several ABA-dependent stress response genes (*RD29A*, *RD29B*, *RAB18*, *RD26*, *RD20* and *COR15*) in the transgenic plants increased significantly compared with their expression levels in the wild type plants. Ectopic expression of *FtbZIP5* in *Arabidopsis* can partially complement the function of the ABA-insensitive mutant *abi5-1* (abscisic acid-insensitive 5-1). Moreover, we screened FtSnRK2.6, which might phosphorylate FtbZIP5, in a yeast two-hybrid experiment. Taken together, these results suggest that FtbZIP5, as a positive regulator, mediates plant tolerance to salt and drought through ABA-dependent signaling pathways.

## 1. Introduction

Exposure to environmental stresses such as drought and salt can adversely affect plant growth and crop yield [1]. The phytohormone abscisic acid (ABA), as a component of signaling, is widely involved in the developmental process in plants and responds to environmental stimuli [2,3]. Endogenous ABA increases in plants under osmotic stress, and many genes are activated by the ABA response element (ABRE) in the promoter region [4,5]. Currently, the SnRK2-AREB/ABF pathway is considered to govern major ABA-mediated ABRE-dependent gene expression in response to osmotic stress [6].

Among the large number of bZIP transcription factors identified in *Arabidopsis thaliana*, ABA signal transduction has been extensively studied in group A, which has 13 members [7,8]. Based on phylogenetic analysis, the bZIP proteins with three conservative N-terminal domains can be divided into two categories [9,10]. The ABI5/AtDPBF family genes (*DPBF2/AtbZIP67*, *DPBF4*, *AREB3*, *ABI5* and *EEL*) are expressed in seeds and play an important role in seed maturation and development [11,12]. However, the AREB/ABF family of genes (*AREB1/ABF2*, *AREB2/ABF4*, *ABF1* and *ABF3*) are mainly expressed in vegetative tissues under abiotic stress and participate in plant resistance to abiotic stress [13,14]. After their discovery, it was found that the functions of AREB1/ABF2, AREB2/ABF4 and ABF3 did not completely overlap and that they could form homodimers or heterodimers under osmotic stress and, in this form, could coordinate the regulation of ABRE-dependent gene expression according to ABA signaling [15,16]. Therefore, the functions of AREB/ABF transcription factors show diversity and complexity and have potential for further study.

Studies have shown that AREB/ABF must be activated in an ABA-dependent manner, and the post-transcriptional modification is the phosphorylation by SnRK2 proteins (SNF1-related protein kinases subfamily 2) [17,18]. The conserved phosphorylation site RXXS/T in AREB/ABF was identified as the target site of the SnRK2s and other general Ser/Thr kinases [19,20]. Furthermore, studies have compared triple mutants of AREB/ABF and subclass III SnRK2s, which are involved in stress resistance in *Arabidopsis*, and it has been shown that the downstream genes of the ABA-dependent type genes overlap [14]. Loss of SnRK2 protein kinase family function prevents most of the ABA response [21]. In addition, phylogenetic analysis also supports the supposition that SnRK2-AREB/ABF in plants is ubiquitous and part of a major positive regulatory pathway [16,22].

Here, we isolated *FtbZIP5* in tartary buckwheat (*Fagopyrum tataricum*), identified its molecular characteristics and performed functional replenishment experiments in the *Arabidopsis* mutant *abi5-1*. Further study confirmed the role of FtbZIP5. Specifically, the physiological and biochemical changes and the expression levels of the downstream stress genes in the transgenic and WT (wild type) plants were compared after stress treatment. Furthermore, we also screened the SnRK2 protein kinases interacting with FtbZIP5. The results may lead to a better understanding of the regulatory network in which FtbZIP5 participates.

## 2. Results

### 2.1. Cloning and Molecular Characterization of FtbZIP5

**Figure 1:**
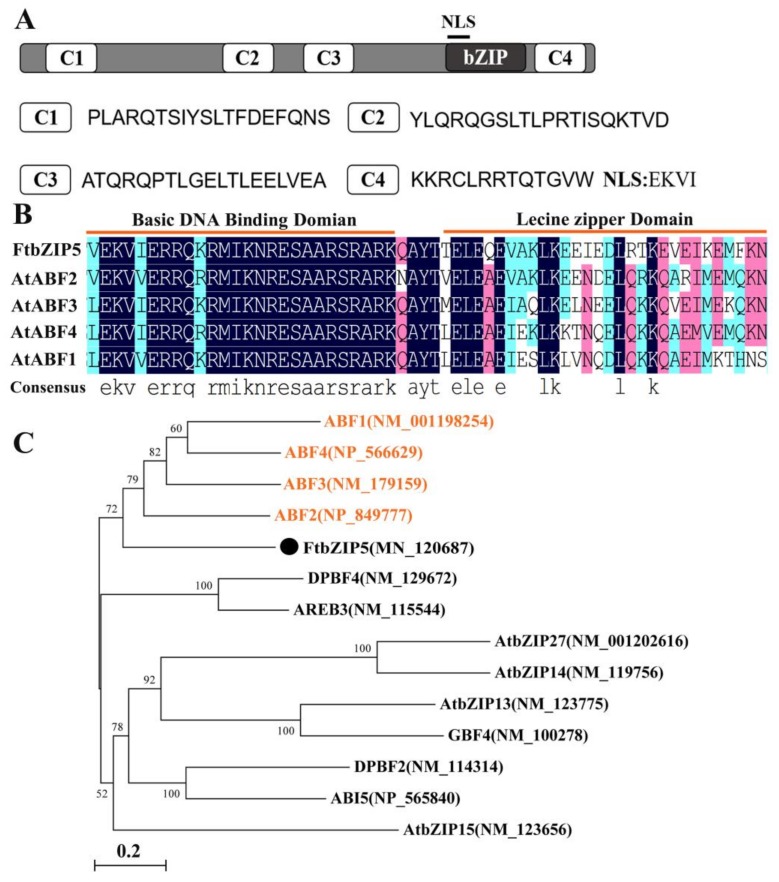
Structure and sequence analysis of FtbZIP5 protein. (**A**) The conserved domain of FtbZIP5 protein. C1, C2, C3 and C4, four potential phosphorylation sites; NLS, hypothetical nuclear positioning signal. (**B**) Sequence alignment of four AREB proteins related to stress resistance in *Arabidopsis*. (**C**) Results from the phylogenetic analysis of FtbZIP5 and members of subfamily A of bZIP transcription factors in *Arabidopsis*.

In this study, *FtbZIP5* (GenBank Accession No. MN_120689) was cloned and isolated from tartary buckwheat. The sequence analysis by ExPASy ProtParam on the online website showed that the ORF was composed of 419 amino acids with a molecular weight of 45.671 kDa and an isoelectric point of 8.46. The results of the multiple sequence alignment analysis show that FtbZIP5 has not only a bZIP domain but also potential phosphorylation peptides, C1, C2, C3 and C4, similar to other identified AREB/ABFs (Figure 1A,B). Furthermore, we chose to construct a phylogenetic tree by combining FtbZIP5 with 13 members of the *Arabidopsis* bZIP transcription factor A subfamily (Figure 1C). The results showed that FtbZIP5 was closely related to four AREB/ABFs involved in stress resistance in *Arabidopsis*.

### 2.2. FtbZIP5 Expression is Involved in the Response to Abiotic Stress

**Figure 2:**
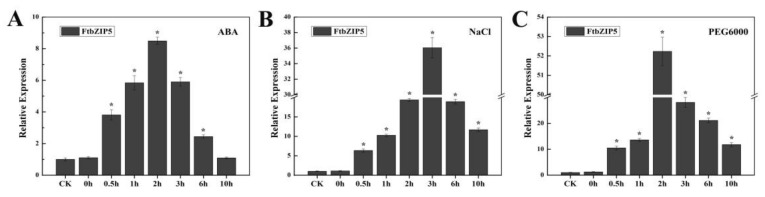
Expression patterns of *FtbZIP5* following ABA (**A**), NaCl (**B**) and PEG6000 (**C**) treatments. The transcriptional abundance of *FtbZIP5* in the absence of a stress treatment was used as the CK (control check). Error bars represent ±SD, and each value represents the averages of three repetitions. Significant difference values were replaced by * to indicate *p* < 0.05.

To further determine whether *FtbZIP5* is involved in the abiotic stress signaling pathway, the expression pattern of *FtbZIP5* after stress was induced was analyzed by qRT-PCR (Figure 2A–C). After 100 μM ABA treatment, the expression level of *FtbZIP5* increased rapidly, more than eight-fold, within two hours and then decreased. However, the changes in *FtbZIP5* expression levels after the salt and drought treatments were more pronounced than those after the ABA treatment and decreased sharply after peaking at 3 h and 2 h, respectively. When treated with 150 mM NaCl and 30% PEG6000 for 3 h, the transcription level of *FtbZIP5* was 35.6-fold and more than 52-fold, respectively. The maximum value (at least eight-fold) was reached after ABA treatment for two hours. The results revealed that *FtbZIP5* might be involved in the response of tartary buckwheat to exogenous stress induced by ABA, drought and salt treatments. Through the analysis of the phylogenetic tree and the expression patterns, it was preliminarily determined that *FtbZIP5* might be involved in the response to abiotic stress.

### 2.3. Characterization of FtbZIP5 as a Transcription Factor

**Figure 3:**
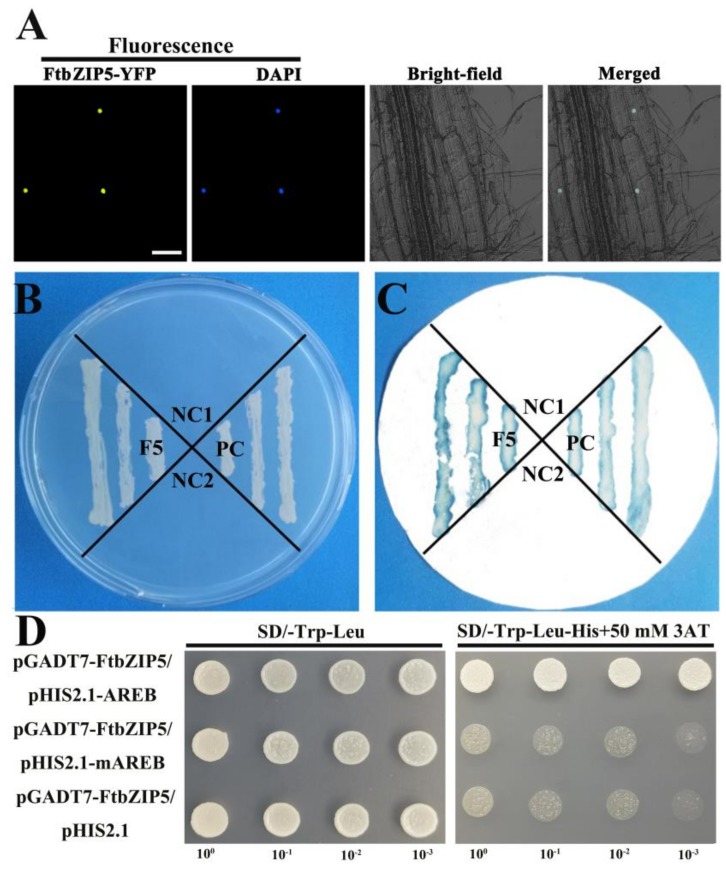
Photographs of FtbZIP5 localization, transcriptional activity and binding to ABRE *cis*-acting elements. Photographs of FtbZIP5 localization and transcriptional activity experiments. (**A**) Subcellular localization of FtbZIP5 protein. Scale bars = 100 μm (**B**) Culture of the transformed yeast cells on SD/-His-Trp medium. (**C**) Experimental analysis of galactosidase staining with x-β-gal on filter paper. NC1:AH109 cells and NC2:pBridge empty plasmids were used as a negative control. PC:pBridge-*FtbHLH2* was the positive control used to evaluate the transcriptional activity of pBridge-*FtbZIP5*. (**D**) Yeast one hybrid experiment with 3×ABRE or 3×mABRE3 as bait. The yeast cells carrying pGADT7-*FtbZIP5* + pHIS2.1-3×ABRE/pHIS2.1-3×mABRE/pHIS2.1 grew on SD/-Trp -Leu and on SD/-Trp-Leu-His containing 50 mM of 3-AT.

To understand the characteristics of FtbZIP5 as a transcription regulator, we determined its localization, transcriptional activity and specific binding elements. As shown in Figure 3A, the yellow fluorescent protein carried by FtbZIP5 was localized identically to the blue fluorescence emitted by DAPI. Furthermore, we examined the transcriptional activation of FtbZIP5 in yeast. All transformants were cultured on SD/-His-Trp plates. The negative control (empty yeast and empty yeast + empty vector) could not grow on the double-deficient medium. The positive control (pBridge-*FtbHLH2*) and pBridge-*FtbZIP5* grew normally on the SD/-His-Trp medium (Figure 3B) and was stained blue by X-β-gal on filter paper (Figure 3C). The results showed that FtbZIP5 had transcriptional activity in yeast. As shown in Figure 3D, all the transformants could grow normally in SD/-Trp-Leu, and only the transformants carrying pGADT7-*FtbZIP5* + pHIS2.1-3×ABRE could grow on SD/-Trp-Leu-His medium containing 50 mM 3-AT. The results proved that FtbZIP5 could be specifically combined with ABRE in a yeast system.

### 2.4. Ectopic Expression of FtbZIP5 Increased the Sensitivity of Arabidopsis Seeds to Exogenous ABA at the Germination Stage

**Figure 4:**
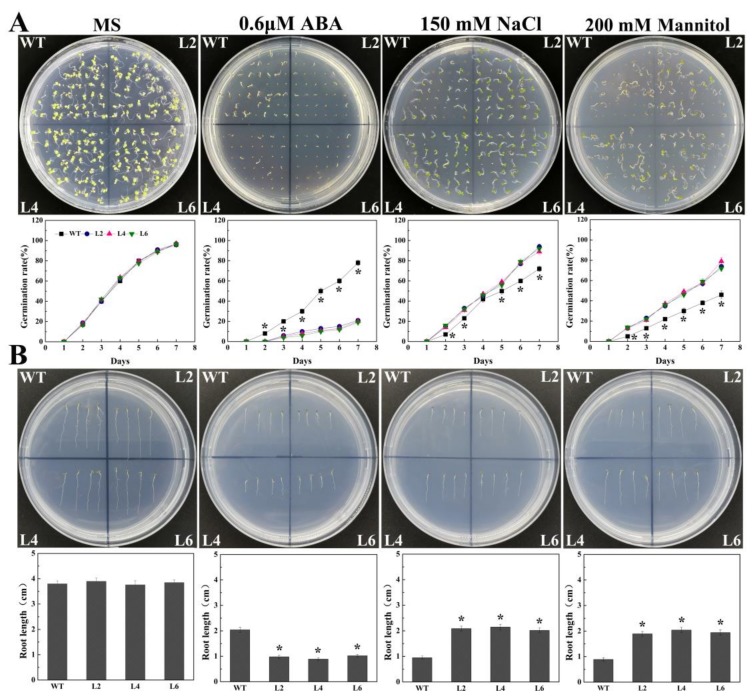
Comparison of the effects of abiotic stress on seed germination and root elongation of WT plants and of the transgenic lines. (**A**) The germination rate of the WT plants and transgenic line seeds cultured on 0.6 μm ABA, 150 mM NaCl and 200 mM mannitol in 1/2-strength MS medium for 7 days. (**B**) The phenotype and root length of transgenic and WT plants were analyzed on 1/2-strength MS plates with 0.6 μm ABA, 150 mM NaCl and 200 mM mannitol for 10 days. The growth of the WT plants and transgenic lines in the 1/2-strength MS plates was used for control values. The means ± SD represent each value from three replicate experiments. * Indicates a significant difference between the transgenic lines and the WT plants (*p* < 0.05).

To explore the effect of ectopic expression of *FtbZIP5* during the resistance of transgenic *Arabidopsis*, we analyzed the germination rate and root elongation of the transgenic lines (L2, L4 and L6) and the WT seeds under adverse conditions. In 1/2-strength MS medium without stress conditions, the germination rates and the root lengths of the transgenic lines and the WT were similar at 7 days. However, in 1/2-strength MS medium containing 0.6 μM ABA, the germination rate of the WT seed was at least three-fold that of the transgenic line on the 7th day (Figure 4A,B**)**. In addition, in 1/2-strength MS medium containing 150 mM NaCl or 200 mM mannitol, the germination rates and root lengths of the transgenic lines were significantly greater than those of the WT. These results showed that the ectopic expression of *FtbZIP5* decreased the sensitivity to salt and drought and increased the sensitivity to exogenous ABA.

### 2.5. Ectopic Expression of FtbZIP5 Enhanced the Resistance of Transgenic Arabidopsis to Drought and Salt Stress

**Figure 5:**
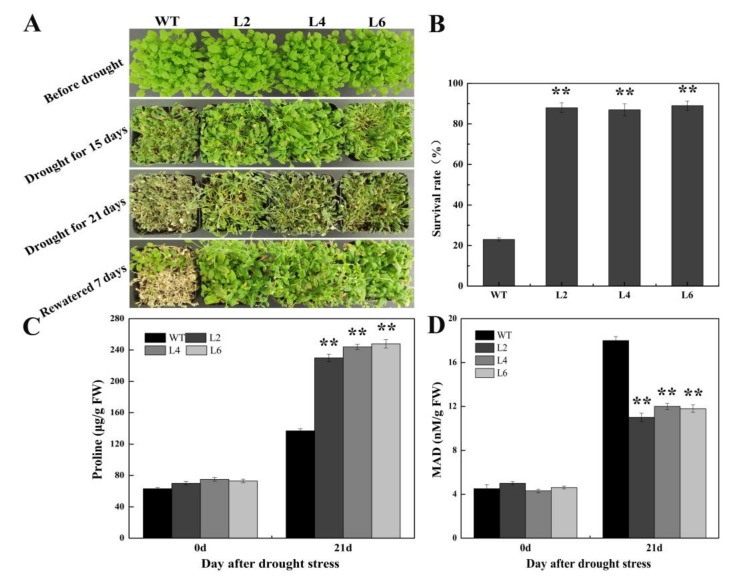
Photographs showing a comparison of the drought resistance state of the transgenic and WT plants. (**A**) Effects of drought on the transgenic and WT plants. (**B**) The survival rate of the transgenic and WT plants after being rewatered for 7 days. (**C**) The proline content. (**D**) The MDA content. Error bars represent ±SD, and each value represents the averages of three repeated measurements. ** indicate significant differences between the transgenic plants and the WT plants (*p* < 0.01).

**Figure 6:**
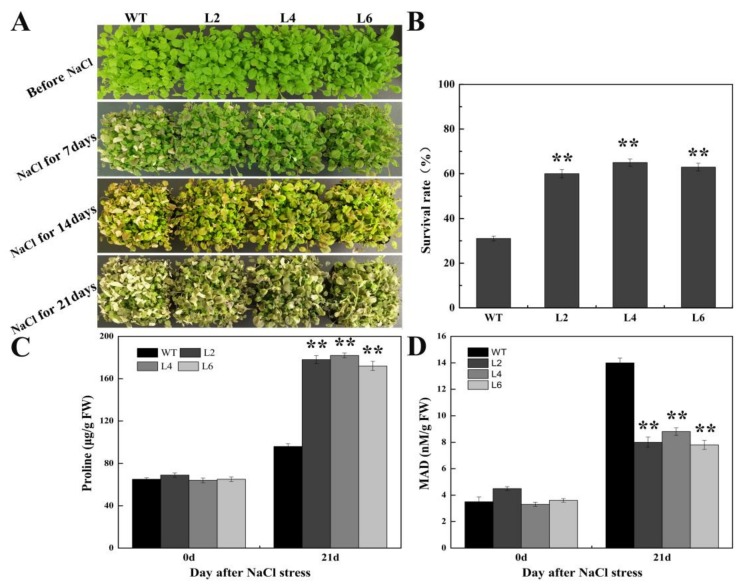
Photographs showing a comparison of the salt tolerance between transgenic and WT plants. (**A**) Effects of drought on the phenotypes of the L2, L4, L6 and WT plants. (**B**) The survival rate was calculated 7 days after rehydration. (**C**) Proline content. (**D**) MDA content. Error bars represent ±SD, and each value represents the averages of three repeated measures. ** indicate the significant differences between the L2/L4/L6 plants and the WT plants (*p* < 0.01).

**Figure 7:**
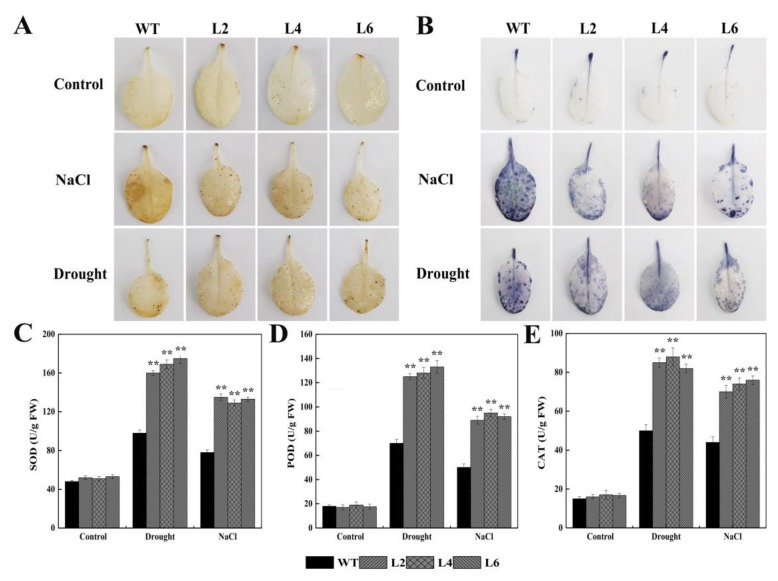
Effects of ectopic expression of *FtbZIP5* on ROS accumulation and oxidative damage in *Arabidopsis*. The accumulation of H_2_O_2_ and O_2_^-^ in the L2, L4, L6 and WT leaves were analyzed by DAB (**A**) and NBT (**B**) staining. (**C–E**) Determination of SOD, POD and CAT activity. The value is the mean ± SD of three biological replicates. ** Indicates significant differences between the L2/L4/L6 plants and the WT plants. ** Indicates *p* < 0.01.

**Figure 8:**
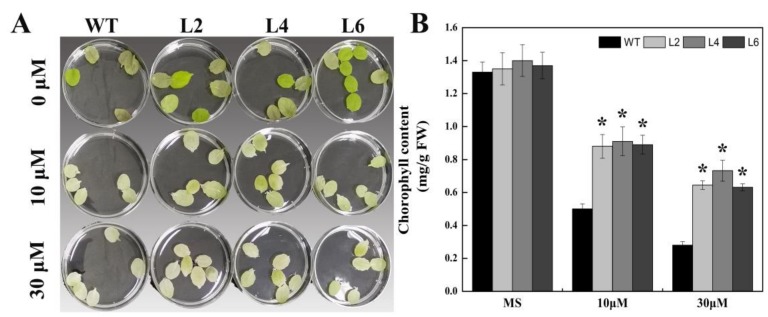
Comparison of the oxidative stress tolerance between the WT and the transgenic plant leaves. (**A**) The leaves of three-week-old L2, L4, L6 and WT *Arabidopsis* were treated with methyl viologen (MV) at concentrations of 0, 10 and 30 μM for 24 h. (**B**) Chlorophyll content in the leaves after stress treatment. Each value is based on three biological replicates, and the error bars represent ±SD. *Indicates significance compared with the WT (*p* < 0.05).

To confirm the function of FtbZIP5 in *Arabidopsis* stress resistance, the phenotypic and physiological changes in the transgenic lines and the WT were analyzed after drought, salt and oxidative stress. The initial number of plants used before treatment was 30 plants per pot, and the survival rate of transgenic lines (L2, L4 and L6) after 3 weeks of dewatering and 7 days of rewatering was approximately 60% higher than that of WT (Figure 5B). In addition, the survival rate of the WT plants was at least 25% lower than that of the L2, L4 and L6 plants after 3 weeks of salt stress treatment. Furthermore, the levels of the antioxidant enzymes (SOD, POD and CAT) and the proline content in the two-week-old transgenic subjected to drought conditions or treated with salt were more insensitive than those of the WT plants (Figure 5C, Figure 6C, Figure 7C–E), while the content of MDA was more insensitive than that of the WT (Figure 5D, Figure 6D). Consistently, the results showed that the chlorophyll degradation rate in the transgenic lines was lower than that of the WT at MV concentration of 10 μM and 30 μM MV, as shown in Figure 8A,B. 

To investigate whether FtbZIP5 regulates ROS balance in plants, we quantitatively measured ROS (H_2_O_2_ and O2^-^) accumulation in the L2, L4, L6 and WT leaves by DAB and NBT staining after 10 days of drought or salt treatment. As shown in Figure 7A and 7B, the ROS accumulation in the transgenic plants was significantly lower than that in the WT plants. In conclusion, our results suggest that FtbZIP5 may enhance the stress resistance of transgenic *Arabidopsis* by regulating the accumulation of ROS and inducing antioxidant systems in plants.

### 2.6. Ectopic Expression of FtbZIP5 Regulated Downstream Stress Response Genes

**Figure 9:**
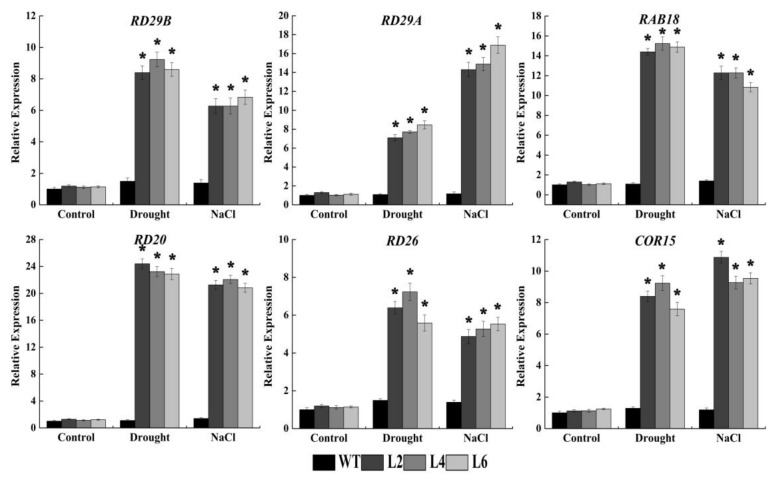
Analysis of ABA-dependent stress-related gene expression levels in transgenic lines and WT. Three-week-old *Arabidopsis* seedlings were treated without water and with 200 mM NaCl for one week until the RNA was extracted. The expression level of the WT under normal growth conditions was used as a control. The error bar represents the standard deviation between the three biological replicates. * Indicates that the expression level of the gene in the transgenic lines was significantly different from that in the WT. Significance was based on Student’s t-test (*p* < 0.05).

To study the molecular mechanism of FtbZIP5 regulation of stress response-related genes, qRT-PCR was used to detect the expression levels of ABA-dependent stress response genes, including *RD29A*, *RD29B*, *RAB18*, *RD26*, *RD20* and *COR15,* in *Arabidopsis* under drought or salt stress. The expression level of the transgenic lines (L1, L3 and L5) and WT plants without treatment was used as the corresponding control, and as shown in Figure 9, there was no remarkable change. However, after the drought or salt stress treatment, the expression of these stress response genes increased significantly. Thus, we speculate that FtbZIP5 might regulate stress-related genes through the ABA signaling pathway to increase plant resistance to drought exposure and high salt levels.

### 2.7. Ectopic Expression of FtbZIP5 Partially Recovered the Insensitivity of the Mutant abi5-1 to ABA

**Figure 10:**
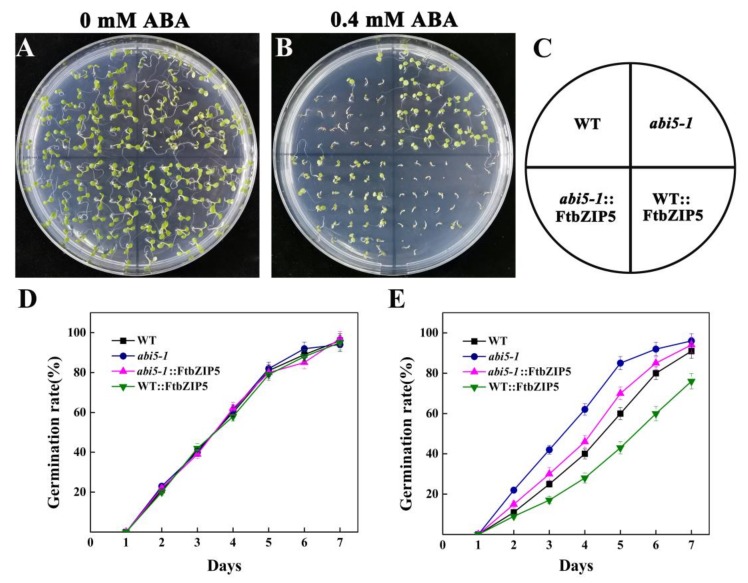
Ectopic expression of *FtbZIP5* increases the ABA sensitivity of *abi5-1* in *Arabidopsis* during seed germination. (**A**,**B**) The seeds of the WT, *abi5-1*, *abi5-1*:: FtbZIP5 and WT:: FtbZIP5 plants were cultured on 1/2-strength MS medium with 0 μM ABA or 0.4 μM ABA for 7 days. (**C**) A schematic map showing the four areas sown on the medium. (**D**,**E**) The germination rate of the seeds was calculated.

To determine whether FtbZIP5 has biological significance in the ABA signaling pathway in the plants, we constructed *abi5-1* transgenic lines from pCAMBIA1305-*FtbZIP5* (*abi5-1*::FtbZIP5). Seed germination of *FtbZIP5*-overexpressed *Arabidopsis* lines (WT::FtbZIP5), WT, mutant abi5-1 and abi5-1 were compared by 0 μM ABA and 0.4 μM ABA treatment. As shown in Figure 10A and 10D, the germination rates of the different types of seeds were basically the same on the 7th day when none were treated with ABA. When 0.4 μM ABA treatment was used, the germination time and rate of the mutant *abi5-1* seeds were similar to those under normal conditions, and the germination time and rate were inhibited by ABA in the other seeds (Figure 10B,E). Surprisingly, the seed germination of the *FtbZIP5*-overexpressed lines (WT::FtbZIP5) was inhibited most obviously, and the seeds of the WT and *abi5-1* transgenic lines were moderately inhibited. Our results revealed that *FtbZIP5* was able to partially compensate for mutant *abi5-1* during seed germination.

### 2.8. The Physical Interaction between FtbZIP5 and SnRK2.6 Proteins in Yeast

**Figure 11:**
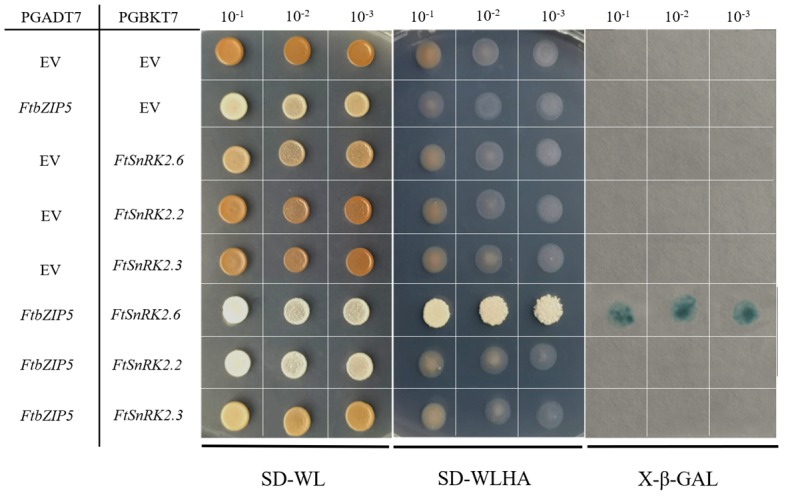
Y2H experiment used to analyze the relationship between FtSnRK2.6/2.3/2.2 and FtbZIP5. The combination plasmid transformed in AH109 yeast cells were serially diluted 10-, 100- and 1000-fold, cultured on SD/-Trp-Leu medium and SD/-Trp-\Leu-His-Ade medium, respectively, and then stained with x-β-gal.

Based on three members of the third subclass of FtSnRK2 protein kinases obtained from tartary buckwheat by previous studies [23], the interaction analysis was carried out by a yeast two-hybrid experiment. Both the negative control and experimental transformants could grow normally on the double-deficient medium. However, only the FtSnRK2.6 + FtbZIP5 yeast strain survived in the four deficient media and showed blue color indicative of x-β-Gal on filter paper (Figure 11). Collectively, these data allowed us to conclude that FtbZIP5 and FtSnRK2.6 can interact with each other.

## 3. Discussion

**Figure 12:**
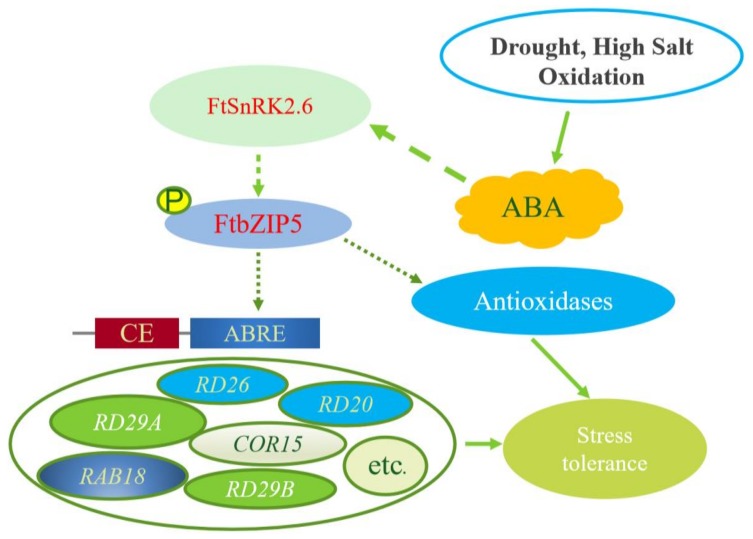
FtbZIP5 of *Fagopyrum tataricum* is involved in the ABA-dependent signaling pathway and may improve the stress tolerance of plants via the phosphorylation of FtSnRK2.6. Dotted and dashed lines indicate that the relationship between the two is not directly proved, while complete arrow lines indicate that it has been confirmed.

In this study, we identified a novel stress-resistant AREB/ABF transcription factor gene, FtbZIP5, from tartary buckwheat. Similar to other studies, the protein is located in the nucleus, has transcriptional activation activity and can specifically bind to ABRE *cis*-acting elements. Similar to other studies, the protein is located in the nucleus and has transcriptional activation activity. The results from the bioinformatics analysis showed that FtbZIP5 has a high affinity for ABFs related to stress in *Arabidopsis* and had four possible phosphorylation sites. Additionally, the results from the expression analysis showed that *FtbZIP5* was significantly induced by drought, salt and exogenous ABA. Further studies showed that the ectopic expression of *FtbZIP5* could regulate stress-induced genes through an ABA-dependent signaling pathway and enhance drought and salt tolerance in transgenic *Arabidopsis* by increasing SOD, POD and CAT levels, thereby reducing the ROS content of transgenic *Arabidopsis*. Furthermore, the ectopic expression of *FtbZIP5* can increase the antioxidant activity and sensitivity of transgenic plants to exogenous ABA and partially restored the insensitivity of mutant *abi5-1* to ABA. Additionally, we also screened FtSnRK2.6, which may phosphorylate FtbZIP5. Based on the above results, we propose a diagram showing the role of FtbZIP5 in the stress response regulation mechanism (Figure 12). This study can be used to advance the understanding of the tartary buckwheat stress resistance mechanism and enrich the research into AREB/ABFs in plants.

Previous studies have demonstrated that AREB/ABFs, as positive regulators of the ABA signaling pathway, play important roles in osmotic stress regulation in plants [24]. For example, overexpression of *OSBZIP72* from rice enhanced the drought resistance and ABA sensitivity of transgenic plants [25]. The wheat TaAREB3 transcription factor improved the drought and freezing tolerance in *Arabidopsis* [26], and overexpression of GhABF2 enhanced the salt and drought tolerance of cotton [27]. In *Arabidopsis*, the homologous protein ABI5 of the AREB/ABF family is the central regulator of ABA signal transduction [28,29]. Previous studies have shown that mutants of ABI5 and members of the AREB/ABF family reduce the insensitivity to exogenous ABA during seed germination [30,31]. Our results showed that the ectopic expression of *FtbZIP5* increased the sensitivity of mutant *abi5-1* to ABA. However, the ectopic expression of *FtbZIP5* could not make the *abi5-1* mutant reach the same sensitivity to ABA as the WT. Similar to the findings from previous studies, the functions of ABI5 and AREB/ABFs are redundant, and ABI5 plays a major regulatory role within 10 days of seed germination [32].

It is well known that plants can undergo a series of physiological and biochemical adjustments in vivo to adapt to adverse living environments. Reactive oxygen species (ROS) accumulate in plants under abiotic stress, and excessive ROS can cause oxidative damage to cell components [33,34]. Plant scavenging enzyme (SOD, POD and CAT) systems can remove redundant ROS, thereby increasing plant adaptability [35]. Malondialdehyde (MDA) is the most important product of membrane lipid peroxidation [36]. Proline acts as an osmotic regulator in plant cytoplasm [37]. Both MDA and proline are main indicators for determining plant stress resistance. Previous studies have shown that AREB/ABFs, such as ABP9 and VvABF1, can increase antioxidant enzyme activity and proline content, and reduce ROS accumulation and MDA content to improve the resistance of transgenic plants [38,39]. As expected, our experimental results also show that FtbZIP5 has functions similar to those of these AREB/ABFs. Additionally, after activation of the AREB/ABF-SnRK2 signaling pathway, AREB/ABF transcription factors directly interact with ABRE elements in the promoter region of downstream stress response genes [40]. It has been shown that these six stress/ABA-induced genes (*RD29A, RD29B, RAB18, RD26, RD20* and *COR15*) are directly regulated by ABRE/ABFs in *Arabidopsis* [41,42]. The results of yeast one-hybrid experiment also showed that FtbZIP5 could specifically bind to ABRE *cis*-acting element.

Phosphorylation, acetylation, methylation and other post-translational modifications can integrate multiple signals at the same level [21]. In *Arabidopsis*, studies have revealed that triple mutants of snrk2.6, snrk2.3 and snrk2.2 control ABA-activated phosphorylation of AREB/ABFs [43]. Interestingly, subsequent study found that AREB/ABFs were phosphorylated by multiple protein kinases of the SnRK2 family [44]. Recent reports have also suggested that the response of plant cells to salt stress, drought stress and stress hormone ABA-induced signaling depends largely on the SnRK protein kinase family [45]. Our yeast two hybrid experiment showed that there was interaction between FtbZIP5 and the homologous protein FtSnRK2.6 of SnRK2.6, but their phosphorylation requires further verification. Similarly, in apple, MdAREB2 is phosphorylated by the SnRK2 homologous protein MdCIPK22 [46]. Previous studies have found an interaction between FtbZIP83 and FtSnRK2.6/2.3 [23]. The differences in their interaction may be caused by the complexity of AREB/ABF transcription factor function and the diversity of their post-transcriptional modifications. Collectively, the data suggest that the SnRK2-AREB/ABF signaling pathway plays a pivotal role in the cross-talk of that is induced by abiotic stress signaling in plants.

## 4. Materials and Methods

### 4.1. Cloning of FtbZIP5 Gene, Construction of the Vector and Transformation of Arabidopsis Thaliana

To construct the fusion protein expression vector pCHF-*FtbZIP5*-YFP, a pair of specific primers were designed to amplify the cDNA sequence of FtbZIP5. The CDS of FtbZIP5 was cloned into the plant expression vector pCHF-YFP by *BamH*I and *EcoR*I.

The positive *Agrobacterium* contained 35S:*FtbZIP5*-YFP, which was transferred into WT *Arabidopsis* through the floral dip transformation method [47]. Then, the transgenic homozygous cell lines were screened by 1/2-strength MS medium containing 50 mg/mL kanamycin and confirmed at the molecular level by PCR. For transcriptional activation experiments, the CDS of FtbZIP5 was inserted into the pBridge vector to generate pBridge-*FtbZIP5* using the two restriction sites: *EcoR*I and *Sal*I. In the yeast one hybrid assay, the three tandem copy sequences of ABRE (ACGTG) and mABRE (AAAAA) were inserted into pHIS2.1 vector of *EcoR*I and *BamH*I sites, respectively. Using *Nde*I and *EcoR*I as restriction sites, the CDS of FtbZIP5 was cloned into the vector pGADT7. For the functional replenishment mutant experiment, we constructed a vector, pCAMBIA1305-*FtbZIP5*, that was transferred into *Arabidopsis* mutant *abi5-1* via *Agrobacterium*-mediated transformation. The homozygous transgenic lines of generation T3 were obtained by the methods described above. The primers are presented in Supplementary Table S1.

The phylogenetic tree was constructed using the neighbor-joining method with Mega 7.0 software, and DNAMAN software was used for multiple sequence alignment. The physicochemical properties of FtbZIP5 protein were obtained through ProtParam.

### 4.2. Plant Materials and Stress Treatments

The tartary buckwheat seeds (Xiqiao No. 2) used in this study were provided by the Academy of Agricultural Sciences (Xichang, China). The seeds were germinated on wet filter paper and then transferred to Hoagland solution for culturing. For the stress treatment, 2-week-old seedlings with similar growth and development were treated with 100 μM ABA, 30% PEG and 150 mM NaCl. Samples were collected at 0, 1, 1.5, 2, 3, 6 and 10 h of treatment within a photoperiod and then stored immediately at −80 °C until the RNA was extracted. 

For the seed germination experiments, sterilized WT and transgenic seeds were germinated on 1/2-strength MS agar plates containing 0.6 μM ABA, 150 mM NaCl and 200 mM mannitol. The germination rate of the seeds was recorded every 24 h for 7 days. For the root length experiments, seedlings aged 3 days and growing in 1/2-strength MS media were transplanted to the same stress plate as described above, and the root length was measured on the 7th day. The complement mutant experiment was carried out with 0.4 μM ABA, which was similar to that used in the seed germination experiment.

For the drought and salt stress experiments, the T3 homozygous transgenic lines and WT plants were cultivated in soil for three weeks under the same growth conditions. For the drought treatment, 3-week-old seedlings were denied water for 2 weeks and then were rewatered to determine the survival rate 7 days after the rewatering was initiated. For the salt treatment, 3-week-old seedlings were treated with 200 mM NaCl for 3 weeks, and their phenotypic changes were recorded every 7 days.

For the oxidative stress experiment, the leaves from the 4-week-old transgenic lines and the WT were treated with 1/2-strength MS containing 10 µM or 30 µM methyl viologen, respectively. After 48 h, the degree of chlorosis was observed, and the chlorophyll content was determined according to the Heath et al. method [48]. The abovementioned growth conditions were generated with a white fluorescent lamp (14 h/10 h, light/dark) at room temperature of 23 ± 1 °C with relative humidity of 50%–60%.

### 4.3. RNA Extraction and qRT-PCR Analysis

Total RNA from mixed tissues of the plant seedlings was extracted using an EASYSpin Plant RNA kit (Aidlab, Beijing, China), and the first-strand cDNA was synthesized by being reverse transcribed with a HiScript II 1^st^ strand cDNA synthesis kit (Vazyme Biotech, Nanjing, China). The RT-PCR amplification enzyme (TB Green Premix Ex TapII), primer and template were mixed and amplified in a CFX96-RT-PCR machine. Three biological replicates were generated for each sample, and the results were calculated by the 2^−ΔΔ*C*T^ method. The primers used for qRT-PCR are listed in Supplementary Table S2.

### 4.4. Subcellular Localization, Transcriptional Activation and Binding with ABRE Elements of FtbZIP5 

For the localization study, the FtbZIP5:YFP fusion protein was selected for transformation into *Arabidopsis* using a method similar to that of previous studies [49]. The sterilized seeds of transgenic *Arabidopsis* were cultured in 1/2-strength MS for two weeks. DAPI, as a marker of nuclear positioning, emits blue fluorescence under blue excitation light. The results were obtained by observing the mature zone of the roots with yellow and blue excitation light in confocal laser microscopy. For the transcriptional activation assays, pBridge-*FtbZIP5*, pBridge-*FtbHLH2* and pBridge (negative control) were transformed into yeast AH109. A galactosidase activity assay was performed on a filter paper by selecting transformants that normally grow in SD/-His-Trp media. For yeast one hybrid experiment, pGADT7-*FtbZIP5* + pHIS2.1-3×ABRE/pHIS2.1-3×mABRE/pHIS2.1 vector combinations were respectively transformed into yeast Y187. After 2–3 days of SD/-Trp-Leu culture, the growth was observed on the SD/-Trp-Leu-His medium containing 50 mM 3-AT.

### 4.5. Measurement of Antioxidant Enzymes, Proline and Malondialdehyde

To further understand the function of FtbZIP5 under drought/salt treatment, we measured the activity of three antioxidant enzymes (SOD, POD and CAT) and the Pro and MDA content. After two days of drought and salt treatment, the activity levels of SOD, POD and CAT in the leaves were determined by the method described previously [32]. For the determination of Pro (proline) and MDA (malondialdehyde) content, the transgenic lines and the WT were selected one week after exposure to drought/salt stress, as described previously [50].

### 4.6. Histochemical Staining with DAB and NBT

To evaluate the extent of ROS (H_2_O_2_ and O_2_^−^) accumulation in transgenic lines and the WT after drought and salt stress, DAB and NBT histochemical staining were used. After two weeks of stress treatment, the leaves were stained with NBT (0.5 mg/mL) for 3 h and DAB (1 mg/mL) for 8 h under dark conditions. Then, the stained leaves were boiled in 80% ethanol until the green was completely faded.

### 4.7. Y2H Assay

Yeast two-hybrid (Y2H) analysis was carried out to determine whether FtbZIP5 protein could interact with FtSnRK2.6/2.3/2.2 kinase. The coding sequences of FtbZIP5, FtSnRK2.6, FtSnRK2.3 and FtSnRK2.2 were cloned into pGADT7 or pGBKT7 vectors, and FtSnRK2.6/2.3/2.2 + FtbZIP5 in the experimental group and FtSnRK2.6/2.3/2.2 + pGADT7, pGBKT7 + FtbZIP5 and pGBKT7 + pGADT7 in the negative control group were established. Next, the recombinant plasmids in the above combinations were transferred into yeast AH109. The specific designed primers are shown in Supplementary Table S3. The positive transformants were diluted 0.1-, 0.01- and 0.001-fold, respectively, and then cultured on SD/-Trp-Leu and SD/-Leu-Trp-His-Ade selective media at 28 °C for 3 days. In the last step, yeast normally growing in the four deficient media were stained with X-β-gal on filter paper.

## Supplementary Materials

Supplementary materials can be found at https://www.mdpi.com/1422-0067/21/3/1123/s1.
